# Fabrication of dialysis membrane from cotton Giza 86 cellulose di-acetate prepared using Ac_2_O and NiCl_2_ as a new catalyst

**DOI:** 10.1038/s41598-023-29528-6

**Published:** 2023-02-08

**Authors:** Safaa Ragab, Amany El Sikaily, Ahmed El Nemr

**Affiliations:** Environment Division, National Institute of Oceanography and Fisheries (NIOF), Kayet Bey, Elanfoushy, Alexandria, Egypt

**Keywords:** Chemistry, Biochemistry, Catalysis, Polymer chemistry

## Abstract

This attempt has been made to synthesize the cellulose di-acetate in a solvent-free acetylation system of cotton Giza 86 cellulose with Ac_2_O (200 and 300 ml) in the presence of NiCl_2_^.^6HO (1.0, 1.5 and 2.0 g) as an effectively available and new catalyst by the conventional reflux and microwave irradiation methods. This study also illustrates the preparation of a dialysis membrane made from a cellulose di-acetate–dichloromethane-methanol-polyethylene glycol (MW: 200) casting solution. The microwave irradiation method for the synthesis of cellulose di-acetate showed excellent yields and short reaction time, which is an important feature of this method. The impact of the two methods on the cellulose di-acetate formation and its used in the dialysis membrane formulations was studied. The experimental degree of substitution of the prepared cellulose di-acetate values (DS = 2.00–2.7) showed an agreement with the calculated values by FTIR and ^1^H-NMR analysis methods. The formation of cellulose di-acetate with percentage yields varied from 62.85 to 89.85%. The applicability of the prepared membrane in dialysis operation was evaluated in terms of urea clearance, rejection of Bovine Serum Albumin (BSA) and flux of pure water. Characterization of cellulose di-acetate was achieved through ^1^H-NMR, FTIR, TGA, and BET analyses. The CA-PEG blend membrane was examined by contact angle measurement, porosity, and water uptake of the membrane. The cellulose acetate membrane surface morphology was determined using SEM. It is observable that the fabricated CA-PEG blend membrane from synthesized cellulose di-acetate by using Nickel chloride as a catalyst is showing remarkable rejection of BSA and urea clearance up to 100 and 67.2%, respectively. The present work is promising and applicable in dialysis membranes.

## Introduction

The evolution of solvent-free catalytic reaction, eco-friendly, economically viable, time of reaction and energy reduced methodology was becoming from our urgent demand to performance the chemical processes. The great importance of the esterification process in organic synthesis was attributed to the production of various useful organic compounds, such as polymers^1,2^. A great number of compounds in living organisms such as proteins, nucleic acids, and cellulose, have been made up of polymers. Moreover, polymers were the main constitutes of the minerals like diamond, quartz, and feldspar, as also as man-made materials as textiles, packaging, plastics, aircraft, construction and rope. Substantial investigation has been carried out in the esterification of cellulose, particularly solvent-free reactions and in the field of catalysis^3–5^. A variety of Lewis acids such as ZnCl_2_^6^, CoCl_2_^7^, and in particular, metal triflates such as Sc(OTf)_3_^8^, Bi(OTf)_2_^9^, Cu(OTf)_2_^10^, and Sn(OTf)_2_^10^ have been found to be operational catalysts for acylation. Also, NiCl_2_ was reported to be used as a catalyst to enhance the conversion and rate of acetylation of phenols, thiols, alcohols, and amines with Ac_2_O^11^. The reducing system of NiCl_2_ and NaBH_4_ efficiently produced the required (*Z*)-alkene-based-modified nucleotides, making NiCl_2_ a highly useful reagent to overcome some issues of over-reduction and/or breakdown^12^. Alonso et al.^13–15^ were synthesized the reducing agents consisting of NiCl_2_^.^2H_2_O, a catalytic amount of arene (naphthalene or 4,4′-di-*tert*-butylbiphenyl: DTTB) and excess powder of lithium. When nickel salts (1.5−2.5 equiv) and naphthalene (17 mol.%) were used, alkenes and alkynes were reduced into alkanes^16,17^. Similar to this, LiAlH_4_ (0.5–1.0 equiv) was also employed to accomplish a stereo- and regioselective hydroalumination of disubstituted acetylene in conjunction with a stoichiometric or catalytic quantity of low-valent transition metal halide NiCl_2_^18^.

Several parameters constrained the conversion and reaction rate of the traditional esterification process. In the conventional esterification reaction, the reactants cannot be miscible, causing a slow kinetics communication between the reactants, so a thin film was formed and subsequently, the mass transfer was limited. To overcome these problems, the reaction is usually achieved by carrying out the reaction under supercritical conditions of high temperature and pressure with or without mixing, agitation, ultra-sonication, etc. In spite of the fact that, these methods were becoming highly efficient, but still cost serious and require high energy, making them uneconomic. The microwave heating method has recently been found to be an acceptable method for improving product yields and reaction rates in the synthetic chemical industry with extensive applications^19^.

The microwave treatment is regarded as a secure method for heating chemical mixtures to high temperatures because the energy of the microwave irradiation is transferred through electromagnetic waves and then results in a temperature that is higher than the medium's average temperature due to the interaction between molecules and microwave energy, which shortens reaction times and requires less energy than traditional heating methods by Motasemi and Ani^20^, and Buchori et al.^21^. However, high reaction temperatures were responsible for the high product yield under these circumstances. As it prevents catalyst contamination seen in other chemical processes, this method has been hailed as green, eco-friendly, safe, and reliable^22^. In the past ten years, several protocols for performing organic reactions, such as the acetylation of alcohols, phenols, thiols, amines, and cellulose in the absence of a solvent, have been reported. These protocols can also be accomplished using a microwave with excellent yields and correct times^23–25^.

A variety of polymers can be used to make dialysis membranes. The majority of these are applied polymers, polyacrylonitrile (PAN), including cellulose acetate (CA), polysulfone (PS), polymethyl methacrylate (PMMA), ethylene vinyl alcohol (EVAL) copolymer, and polyamide^26^. Recently, polymeric semi-permeable CA membranes have been widely used in hemodialysis therapy for patients’ renal failure to separate the protein and uremic toxins based on their molecular weights. The CA membranes have asymmetric structures^27^, solvent resistance, thermostability properties^28^, protein binding, minimizing fouling, good resistance to chlorine and solvent^29,30^, and are cheap and easily available^31^.

Polyvinyl alcohol (PVA) and polyethylene glycol (PEG) have been added to cellulose acetate hemodialysis membranes in order to improve their filtration capacity and biocompatibility^32^. Also, for this purpose^33^, was blending sericin with cellulose acetate to make cellulose acetate/sericin blend membranes to determine the efficiency’s increment of CA membrane in the dialysis process. It is observable that the increase of 7.5% sericin in cellulose acetate remarkably enhances the rejection of BSA and urea clearance up to 96 and 60%, respectively. Polyaziridine or polyetyleneimine (PEI) are blended with CA to change the structure and performance efficiency to get the highest BSA rejection and urea clearance properties^34^. Based on urea and creatinine clearance, the effects of D-glucose monohydrate as an additive and formic acid (FA) as a solvent on the functionality of cellulose acetate dialysis membrane were examined^35^. The effect of different molecular weight PEG additives on cellulose acetate asymmetric dialysis membrane performance in urea clearance was studied^36^. By adding different quantities of monosodium glutamate (MSG) and formic acid, the permeability performance of cellulose acetate hemodialysis membranes was examined. The uremic toxins' increased permeability was caused by an increase in MSG concentration from 2 to 6 weight percent, which was a significant factor in determining the membrane morphologies^37^. Activated carbon (AC), zeolite (ZO), and graphene oxide (GO) were used to create porous cellulose acetate mixed-matrix membranes (MMMs). The potential to use them as hemoperfusion (HP) treatment units for the elimination of uremic toxins such p-cresol (PC) and creatinine (CRT) was investigated^38^.

This study aims to prepare cellulose di-acetate without solvents by acetylating cotton Giza 86 cellulose components using a novel catalyst NiCl_2_ utilizing conventional reflux and microwave irradiation methods. A dialysis membrane constructed of a casting solution of cellulose diacetate, dichloromethane, methanol, and polyethylene glycol (MW: 200) is also produced. ^1^H-NMR, FTIR, TGA, and BET studies were used to characterize the produced membrane and cellulose di-acetate. In expressions of urea clearance, pure water flow, and rejection of Bovine Serum Albumin (BSA) solution, the suitability of the produced membrane in dialysis operation is assessed.

## Material and experimental method

### Materials and chemicals

The cotton Giza 86 was collected from Alexandria local market of Egypt. Sodium hydroxide, sodium hypochlorite, Ac_2_O and EtOH were supplied by Fluka analytical. Dichloromethane and methanol were obtained from (Sigma-Aldrich Co, Germany). Poly ethylene glycol (M.W. 200) was obtained from Acros-organic, USA. NiCl_2_.6H_2_O was received from Universal Fine chemicals PVT-LTD, India. Bovin serum albumin was supplied from (Biowest Co, USA). Urea Kits was used as purchased from Diamond Co, Germany.

### Cellulose isolation

To obtain the cellulose, the cotton Giza 86 was treated with 5 wt % sodium hydroxide solution with solid to liquid ratio of 1:10 (W/V) and heated in water bath at 70 °C for 2 h. The mixture was then filtered and washed with distilled water (dw) until the filtrate was clear. The bleaching process was performed via two steps, firstly, after alkali extraction, sodium hypochlorite (NaOCl) 2% solution was used at ratio of 1:10 (W/V) and heated in a water bath at 70 °C for 2 h, and the bleaching mixture was filtered and washed several times by dw. Bleaching step with NaOCl was repeated two times to purify the cellulose component. Secondly, the bleaching process was done with 5% H_2_O_2_ at a ratio of 1:10 (W/V) at 70 °C for 1 h. The mixture was filtered, washed several times by dw, squeezed and dried in an oven for 48 h at 50 °C.

### Synthesis of CAs

#### Conventional method

20 g of extracted cotton Giza 86 cellulose, acetic anhydride (200 or 300 mL) and 2.0 g of NiCl_2_^.^6H_2_O as a green catalyst were combined in a 500 mL round bottom flask fitted with a condenser. The acetylation was performed under reflux at 140 °C for 48 h. After the reaction was complete, the reaction mixture was allowed to cool to room temperature before 100 mL of ethanol was added and thoroughly mixed in to break down the unreacted acetic anhydride. The product was then thoroughly cleaned with distilled water after being thoroughly washed in ethanol to remove any extra unneeded acetic acid and NiCl_2_ byproducts. The acetylated product was then dried for 24 h in an oven at 50 °C.

#### Microwave method

A mixture of Ac_2_O (200 mL), extracted cotton cellulose (20 g) and different weights of NiCl_2_ (1.0, 1.5, or 2.0 g) as a catalyst was placed into a Teflon cup (400 mL) and then put it into the microwave. The mixture was irradiated for 6, 8, or 10 min under an irradiation power of 1400 W. The Teflon cup was removed from the microwave once the reaction was complete and allowed to cool to room temperature. The Teflon cup's contents were transferred to a 500 mL beaker, and then they were mixed with 100 ml of ethanol for several minutes before filtering. The unreacted acetic acid and NiCl_2_ byproducts were then carefully rinsed from the product with distilled water. Samples were dried for 24 h in a 50 °C oven. The products were weighed in order to calculate the yield percentage and weight growth based on the starting dry cellulose. The difference between the product and starting weights was used to determine the weight increase.

### Determination of CA degree of substitution (DS)

The degree of substitution of the prepared cellulose acetates were measured experimentally and by FT-IR spectra and ^1^H NMR analyses^1–5,24,25^.

### Degree of cellulose acetate polymerization (DP)

With the help of an Agilent Gel Permeation Chromatograph (GPC) using tetrahydrofuran as the solvent, the molecular weight, degree of polymerization, and polydispersity of cellulose acetate were all determined. A standard sample with a molecular weight range between 168 and 3,200,000 was used to calibrate the GPC apparatus.

### Preparation of CA-PEG blend membrane

The casting solution was prepared via dissolving of CA (22.5%) in 100 mL of dichloromethane and methanol (9:1) having a constant PEG (MW: 200) wt % of 0.5. To ensure that the polymers thoroughly mixed to create a homogenous solution, the casting solution temperature was maintained at 80 °C with 4 h of continuous stirring. Then, the casting solution was degassed by using the ultrasonic bath for 4 h to ensure no air bubbles were present. The casting solution was then spread over a dust-free glass plate using an Elcometer 4340 automatic film Application, UK with a 250 μm thickness at room temperature. The cast film was left on for 60 s in order to let the solvent evaporate and complete the phase inversion. The cast film was separated and washed for 30 min. at room temperature in a coagulation bath containing distilled water, inducing an exchange between the solvent and water. The cellulose acetate blend membrane was then transferred to a different container filled with distilled water and ethanol and stored at – 4 °C until it was ready for testing.

### Characterization of CA and CA-PEG blend membrane

FTIR data for cellulose extracted from cotton Giza 86, all synthesized cellulose acetate, and CA-PEG blend membrane were obtained using Bruker VERTEX 70 spectrometer coupled to Platinum ATR unit in the range of 400–4000 cm^−1^. ^1^H-NMR for CA was obtained by 400 MHz Bruker Nuclear Magnetic Resonance Spectrometers and deuterated chloroform as a solvent. Utilizing the SDT650 Simultaneous Thermal Analyzer equipment, TGA measurements for cellulose, CA, and manufactured CA-PEG blend membrane were made in the range of 50 and 600 °C using a 5 °C/min ramp temperature underflow of high-quality N_2_ (100 mL/min) gas. The DSC/TG apparatus was calibrated before tests following the manufacturer's recommendations regarding weight, temperature, and DSC calibrations. Before each run, the instrument was also purged with air and a nitrogen flow for 10 min. By using the BELSORP mini-II, made by BEL Japan, INC, at 77 K as the adsorption temperature and saturated vapor pressure, measurements of the N_2_ adsorption–desorption isotherm under liquid nitrogen temperature were used to determine the specific surface area, pore volume, mean pore diameter, and mean particle size of the synthesized cellulose acetate and fabricated CA-PEG blend membrane. The samples were pre-treated at 120 °C with nitrogen gas flowing through them. The crystallinity and atomic or molecular structure of the synthesized CA and constructed CA-PEG mix membrane were examined using a Bruker D2 PHASER advance XRD analyzer. Using the SEM model JSM 6409A from JEOL, Japan, the surface and cross-section morphology of the manufactured CA-PEG blend membrane was examined.

### Pure water flux

**Figure 1:**
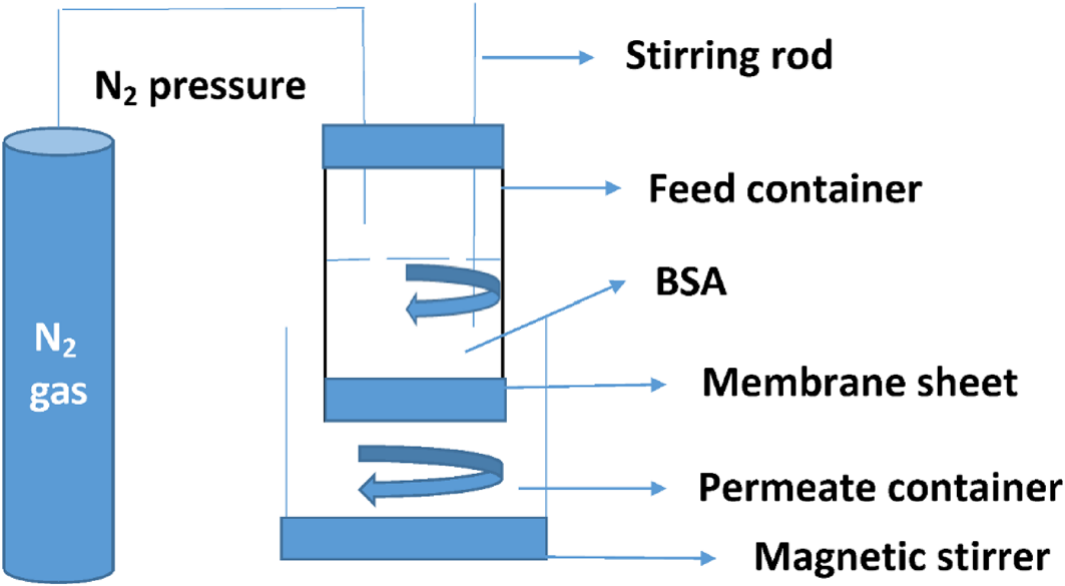
Schematic Figure of dead-end filtration cell for water flux and BSA rejection.

The fabricated CA-PEG blend membrane was cut with an area of 12.57 cm^2^ and placed into the dead-end filtration test cell, which was occupied with 250 mL dw and exposed to the 1.0–2.0 bar pressure to performance the pure water permeation flux (PWP). The experiment was performed at room temperature as shown Fig. (1). The permeation flux of pure water (*J*) was measured from Eq. (1).

1 $$Flux (J)=\frac{Q}{\Delta t\times A}$$

where *J* represents the permeation of pure water flux (L m^–2^ h^–1^), *Q* is the permeate solution volume (*L*), *∆*t is the time of permeation (h), and *A* represents the testing membrane active area.

### Contact angle measurement

To define the hydrophobicity or hydrophilicity of the CA-PEG membrane, the contact angle was measured. A drop of water is placed on a material surface of fabricated CA-PEG blend membrane (4 cm^2^), and the drop forms a dome shape on the surface. The contact angle is the angle produced between the surface and the line perpendicular to the edge of the water drop (wetting angle), which measured by using Theta lite optical tensiometer with a digital video camera based optical tensiometer for measurement of static and surface tension of liquids by pendant drop method, Finland production.

### Water adsorption measurements

To calculate the amount of water absorbed under specific circumstances, water absorption is utilized, so the performance of the prepared membrane in water or humid environment can be determined. For the water absorption test, a membrane sample of 4 cm^2^ was dried in an oven at a set temperature and time (12 h) before being put in a desiccator to cool. The membrane sample was weighted as soon as it had cooled. The membrane sample is subsequently submerged in a water under predetermined circumstances, frequently at 23 °C for 24 h. The sample of the membrane was taken out, dried with a lint-free cloth, and weighed. The membrane water absorption was determined using Eq. (2).

2 $$Water abs \%=\frac{Wet weight-Dry weight}{Dry weight}\times 100$$

### Porosity of membrane

The dry–wet weight method can be used to determine the membrane porosity. The membrane porosity is calculated by using Eq. (3).

3 $$\varepsilon =\frac{WW-DW}{Am \times DPW\times Tm}$$

where *WW* (g) is the wet weight, *DW* is the membrane dry weight, *Am* (cm^2^) is the area of the membrane, DPW is the pure water density, and *Tm* (cm) is the membrane thickness.

### BSA Rejection performance

The efficiency of dialysis membrane was determine in the terms of percent of BSA rejection by using the dead-end filtration test cell with effective membrane area of 12.57 cm^2^, and pressure 1.0–2.0 bar at room temperature, as shown in Fig. 1. The concentration of BSA solution used was 1.0 mg/mL. The concentration of BSA in permeate was analyzed by measuring its absorbance with spectrophotometer (Digital a spectrophotometer (Analytic Jena—SPEKOL1300 UV/Visible spectrophotometer) matched with glass cells of 1 cm optical path) at a wavelength of *λ*_550_ nm using the Biuret Reagent^39^. The rejection of BSA of fabricated CA-PEG blend membrane was determined by Eq. (4).

4 $$\% of BSA rejection=1-\frac{Conc. of permeate}{Conc. of retentate }\times 100$$

### Urea clearance efficiency

**Figure 2:**
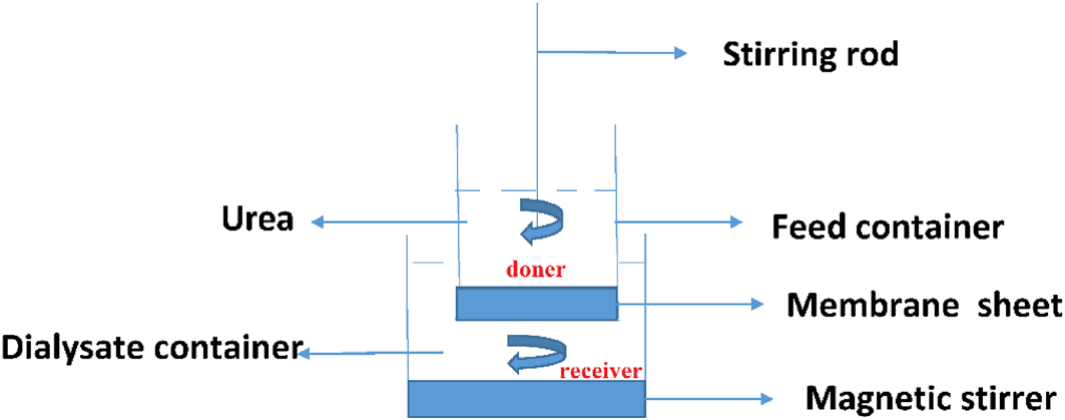
Schematic figure of dialysis cell for urea clearance.

The urea clearance was determined for the fabricated CA-PEG blend membrane. The concentration of urea solution was 50 mL of 1 mg/mL which placed into the donor side and about 2 L distilled water was poured onto the receiver side as shown in Fig. 2. The changing in concentration of urea on donor and receiver sides was measured using enzymatic colorimetric method after every 30 min for 210 min. The concentration of urea was determined by measuring its absorbance with a spectrophotometer at a wavelength of *λ*_578_ nm. The concentration of urea was calculated by using Eq. (5).

5 $$UC\%=\frac{{C}_{0}-{C}_{t}}{{C}_{0}}\times 100$$

where *C*_0_ is the initial concentration at time zero, *C*_t_ is the final concentration at time *t*, and *UC*% is the urea clearance.

## Results and discussion

### The preparation of cellulose acetate

This work is concerned with the preparation of cellulose di-acetate from cotton Giza 86 cellulose, which is convenient for the preparation of dialysis membrane by using a novel Lewis acid catalyst like NiCl_2_^.^6H_2_O and compared between the convention and microwave methods for the acetylation of cellulose. In the conventional method, the effect of amount of acetic anhydride used was only investigated. As shown in Table 1, 20.0 g of cellulose extracted from cotton Giza 86 was refluxed at 140 °C in about 200 mL (Ac_2_O) (Sample 1) and in acetic anhydride (300 mL) (Sample 2) in the existence of 2.0 g of NiCl_2_^.^6H_2_O for 48 h. The previous reactions showed that the % yields of CA were 86.25% (DS = 2.59, Sample 1) and 81.74% (DS = 2.45, Sample 2) with increasing the amount of acetic anhydride from 200 to 300 mL, respectively. So, it has been chosen 200 mL of Ac_2_O and 20 g of cellulose extracted from cotton Giza 86 for acetylation under microwave as an optimum amount. To achieve the targeted DS and control the acetylation reaction conditions, the reaction was repeated under microwave irradiation conditions, changing only the amount of catalyst and reaction time. As reported in Table 1, when the amount of NiCl_2_^.^6H_2_O is 1.0 g, the reaction was performed just after 6 min with a high % yield of 88.30% (31.30 g, DS = 2.63, Sample 3). When the reaction time was extended to 8 min, there was very little change in yields (31.90 g, DS = 2.70, Sample 4) with a percentage yield of 89.85%. While when this reaction was performed for 10 min, the product started to be affected by the time and began to be hydrolysis, so there was a little decrease in the yield (31.20 g, DS = 2.64, 87.94% Sample 5). As shown in Table 1, when the amount of NiCl_2_^.^6H_2_O increased to 1.5 g, the yield was highly decreased to about 23.16, 23.30 and 23.16 g for 6, and 10 min reaction time (DS = 2.0, Samples 6, 7, and 8, respectively). From the previous results, the excess of the quantity of catalyst to 1.5 g was greatly affected the targeted DS values, which became undesirable values (DS ~ 2.6) for the preparation of the dialysis membrane. When increased the quantity of NiCl_2_^.^6H_2_O to 2.0 g as reported in the above-mentioned convention method but under microwave conditions, the product yields were 28.24 g at 6 min, 31.15 g at 8 min, and 24.34 g at 10 min (Table 1). From previous experiments, it can be concluded that, 1.0 g of NiCl_2_^.^6H_2_O as a catalyst using the microwave irradiation method is sufficient to produce cellulose di-acetate with a percentage yield of 89.85% in 8 min in the presence of 200 mL of Ac_2_O.

**Table 1:** Demonstrate conditions and yield of the acetylation of cellulose extracted from cotton Giza 86.

Sample no.	Weight (g)	Time	Ac_2_O (ml)	NiCl_2_ (g)	Yield (g)	CA%	WG (g)	WG FTIR	DS Exp	DS FTIR
1	20	48 h^a^	200	2.0	30.60	86.25	10.6	10.77	2.59	2.65
2	20	48 h^a^	300	2.0	29.00	81.74	9.0	10.26	2.45	2.52
3	20	6 m^b^	200	1.0	31.30	88.30	11.3	11.83	2.65	2.65
4	20	8 m^b^	200	1.0	31.90	89.85	11.9	11.83	2.70	2.69
5	20	10 m^b^	200	1.0	31.20	87.94	11.2	11.28	2.64	2.64
6	20	6 m^b^	200	1.5	23.16	65.27	3.16	3.00	2.00	1.92
7	20	8 m^b^	200	1.5	23.30	62.85	3.30	3.00	2.00	1.91
8	20	10 m^b^	200	1.5	23.16	65.27	3.16	3.00	2.00	1.90
9	20	6 m^b^	200	2.0	28.24	79.59	8.24	8.00	2.39	2.32
10	20	8 m^b^	200	2.0	31.15	87.80	11.15	11.44	2.64	2.66
11	20	10 m^b^	200	2.0	24.34	68.60	4.34	4.00	2.10	1.96

^a^Conventional method.

^b^Microwave irradiation method.

*h* hour, *m* min.

### Characterization of the cellulose acetate and the dialysis membrane

#### FT-IR analysis

**Figure 3:**
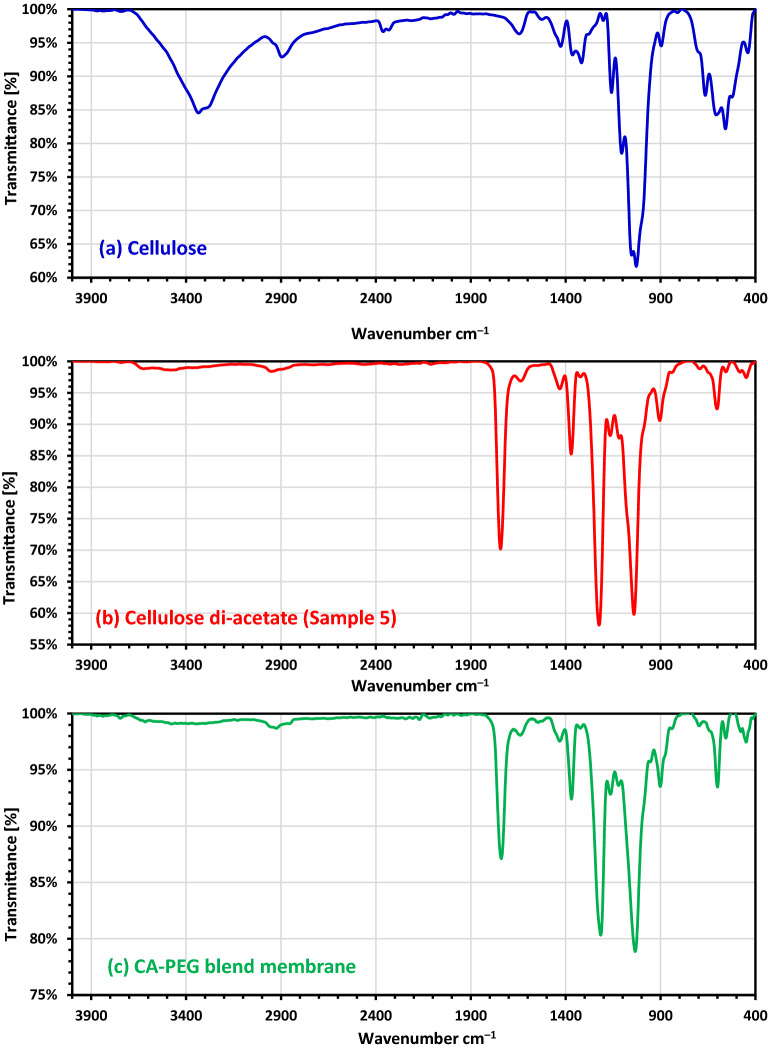
FTIR analysis of (**a**) Cellulose extracted from cotton Giza 86, (**b**) Cellulose di-acetate (Sample-5), (**c**) CA-PEG blend membrane prepared from Sample 5.

All the products (Samples 1–11) were examined by FT-IR analysis, and the degree of substitution (DS) was calculated experimentally and by using FT-IR, the results referred to a successful formation of cellulose di-acetate with different % yields, but also, with a very little variation on DS values (Table 1). The weight gain was also calculated using FT-IR (Table 1). Firstly Sample 5, which has a DS = 2.64 was chosen as a start material for the fabricated CA-PEG blend membrane after testing most prepared cellulose di-acetate samples. Figure 3 displays the FT-IR spectra of cellulose extracted from cotton Giz-86, Sample 5 as a pure cellulose di-acetate and fabricated CA-PEG blend membrane. The FT-IR spectra for the cellulose extracted from cotton Giza 86 displays strong broadband at 3334.81 cm^–1^ due to the stretching –OH, a small band at 2894.73 cm^–1^ ascribed to the stretching mode of –CH, and two bands were greatly decreased in cellulose di-acetate (Sample 5) and in fabricated CA-PEG blend membrane, which proved the successfully performed of cellulose acetylation. Figure 3 shows no chemical change occurred for the pure cellulose di-acetate (Sample 5) in the functionalizing group during blending, except a small shifting was noticed. Figure 3 shows the appearance of strong sharp peaks at 1743.25 and 1739.22 cm^–1^ due to C = O for Sample 5 and CA-PEG blend membrane, respectively. Compared CA (Sample 5) and CA-PEG blend membrane, the absorption peaks appeared sharply at 1370.81 and 1369.39 cm^**−**1^ due to the acetyl group C–H bending band. A strong sharper peak slightly shifted from 1224.01 in Sample 5 to 1215.11 cm^−1^ in CA-PEG blend membrane, which was assigned to the ester C–O stretching band. The peaks at 1040.23 and 1033.56 cm^−1^ belonging to the pyranose ring C–O–C asymmetric stretching were reported for Sample 5 and CA-PEG blend membrane, respectively.

#### ^1^H-NMR analysis

**Figure 4:**
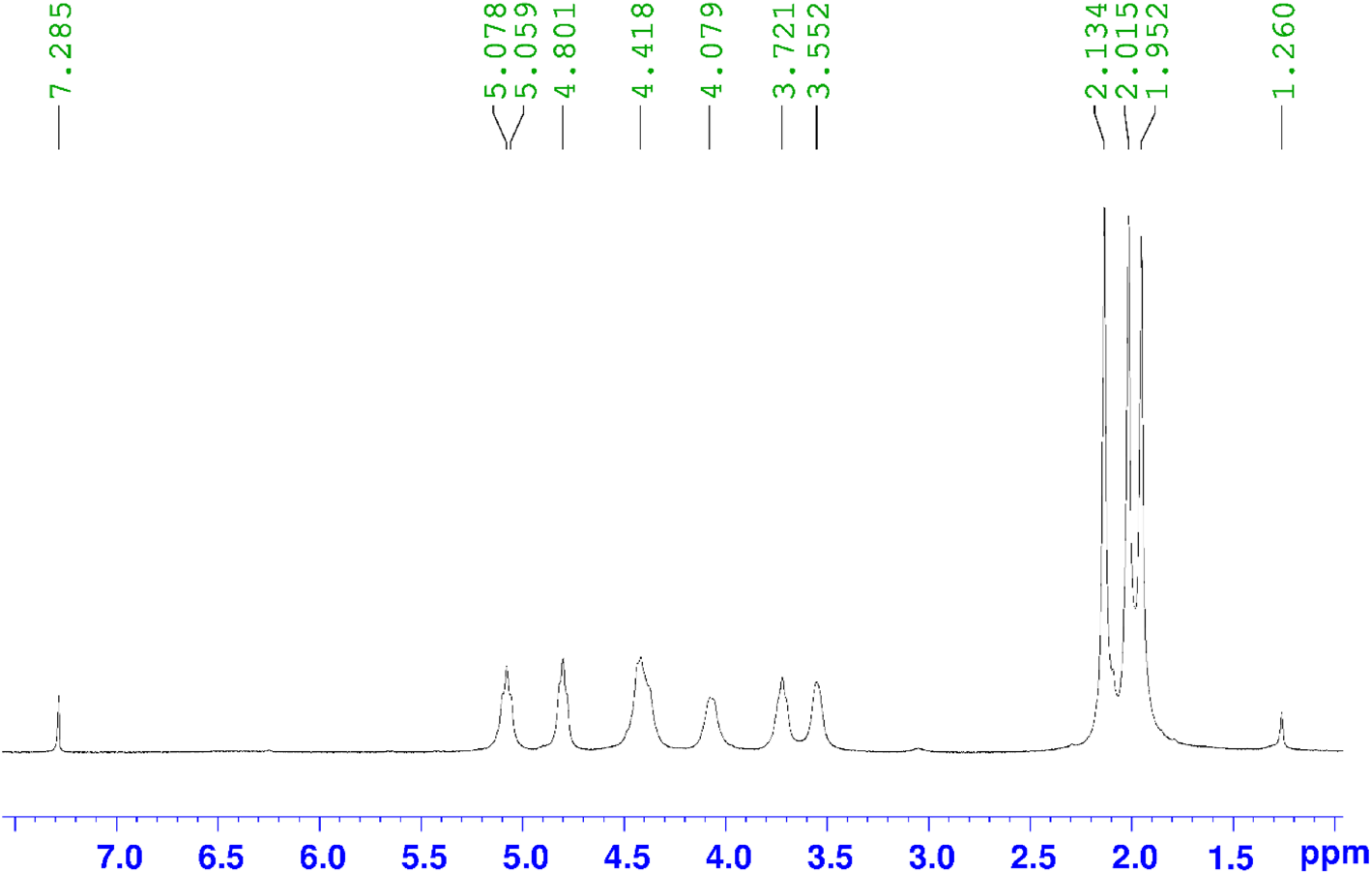
^1^H-NMR spectrum of Sample 5.

The DS of cellulose di-acetate (Sample 5) was measured also by using ^1^H- NMR spectra. Figure 4 displays the ^1^H-NMR spectrum of Sample 5 with a DS value of almost 2.66 demonstrating the creation of cellulose di-acetate using NiCl_2_^.^6H_2_O and microwave irradiation.

#### Degree of polymerization of cellulose acetate (DP)

**Figure 5:**
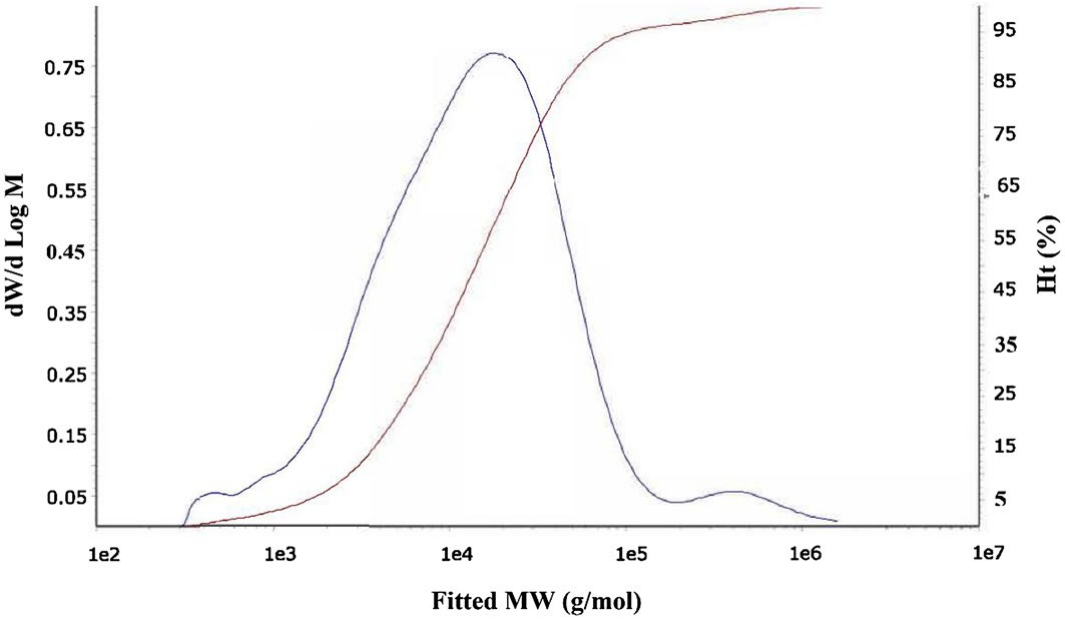
The gel permeation chromatography (GPC) analysis of cellulose di-acetate (Sample 5).

The gel permeation chromatography (GPC) was used to calculate the molecular weight, degree of polymerization, and the polydispersity index of a cellulose acetate (Sample 5) as shown in Fig. 5. From the results, the degree of polymerization was about 149, weight average molecular weight (Mw) was 38,362 (g/mol), number average molecular weight (Mn) was 5864 (g/mol), z average molecular weight (Mz) was 339,278 (g/mol), viscosity average molecular weight (Mv) was 273,659 (g/mol), molecular weight at peak maximum of the elugram (Mp) was 18,098 (g/mol), and the polydispersity index (PD) was 6.542.

#### Thermal analysis

**Figure 6:**
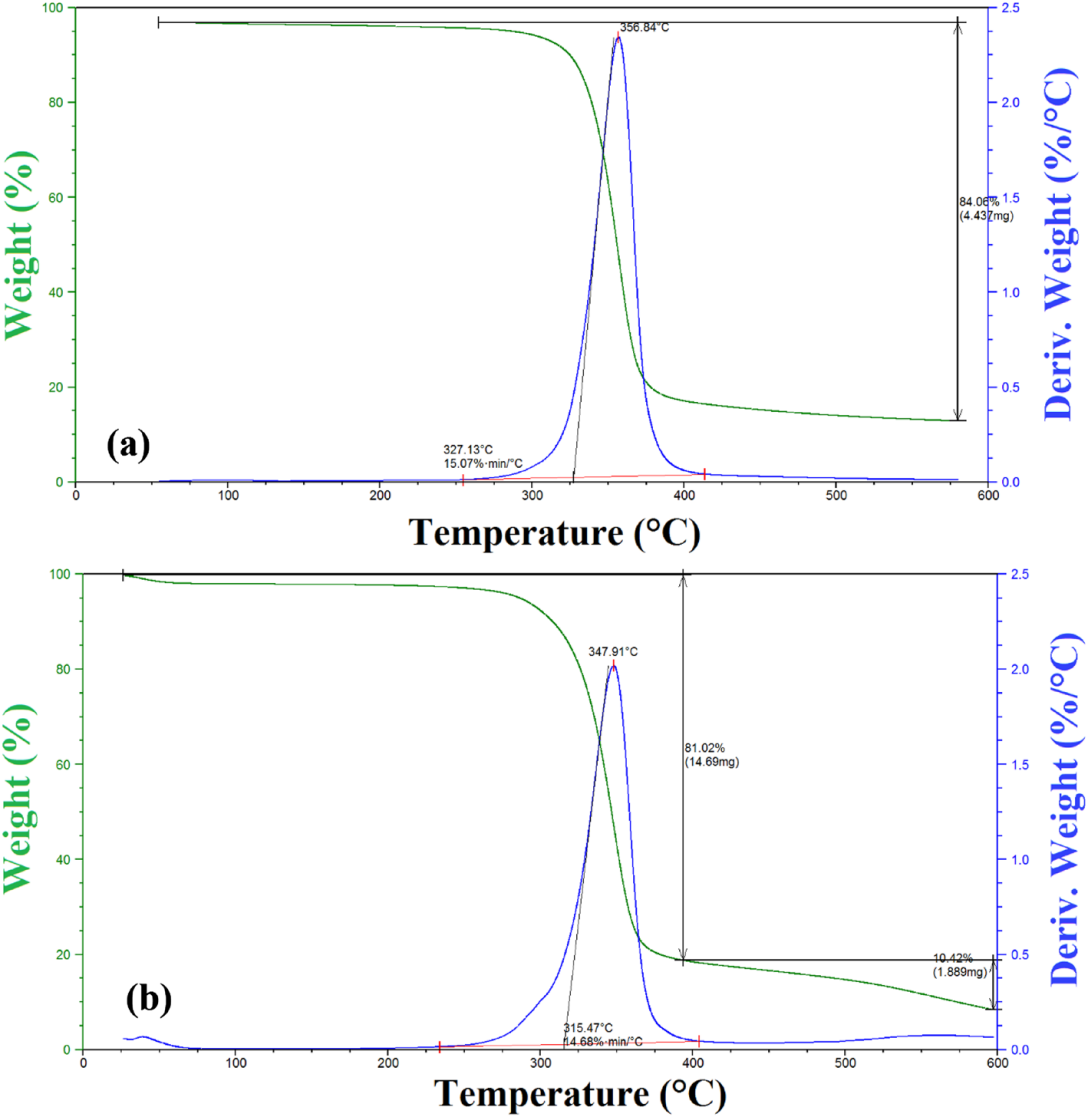
TGA analysis of (**a**) Cellulose di-acetate (Sample 5), (**b**) CA-PEG blend membrane from cellulose di-acetate (Sample 5) and PEG.

Thermo gravimetrical analysis (TGA) is a realistic characterization technique for evaluating the thermal stability of specific materials. The TGA thermal curves of the CA (Sample 5) and fabricated CA-PEG blend membrane are exposed in Fig. 6a,b at a heating rate 5 °C/min. TGA of cellulose di-acetate (Sample 5) (Fig. 6a) shows decomposition occurring via one step and 84.06% weight loss between 327.13 and 600 °C with the maximum decomposition occurring at 356.84 °C. TGA of fabricated CA-PEG blend membrane (Fig. 6b) shows decomposition occurring via two steps. The first weight loss (81.02%) was occurred between 327.13 and 400 °C with the maximum decomposition occurring at 347.91 °C, while the second weight loss (10.42%) was happened between 400 and 600 °C with the maximum decomposition happening at 560.0 °C.

#### Surface area analysis

**Figure 7:**
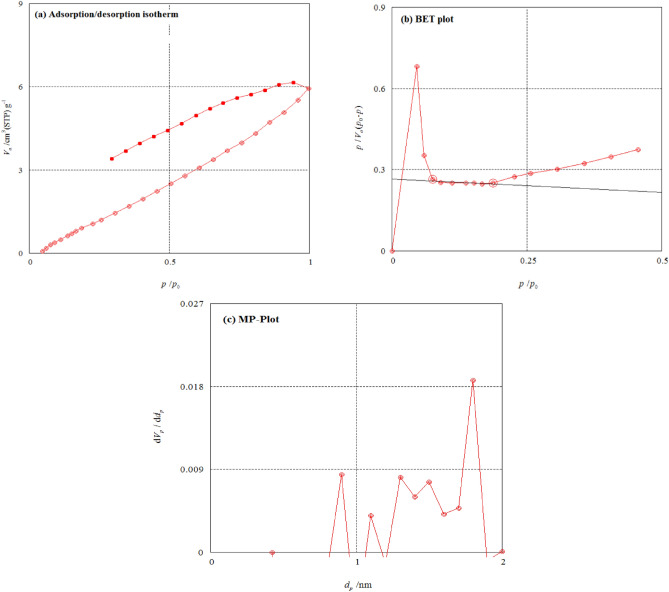
Surface area analysis (**a**) Adsorption/desorption isotherm, (**b**) BET analysis plot, (**c**) Micropore analysis (MP) plot.

BET can produce reliable precision measurement data for the adsorption amount of adsorptive material relative to the pressure. Figure 7a shows the adsorption/desorption isotherm of fabricated CA-PEG blend membrane and also explains the relation between the relative pressure (*p*/*p*_0_) and specific amount adsorbed (*V*_a_: cm^3^ (STP) g^–1^). The results from the Fig. 7b showed that, the adsorption isotherm type may be measured as mixed types of III and V. The monolayer volume (*V*_m_) was 5.9709 cm^3^ (STP) g^–1^, BET specific surface area (*a*_sBET_) was 25.9880 m^2^ g^–1^, energy constant of the first layer (*C*) was 0.6296, total pore volume (*p*/*p*_0_) was 0.00909 cm^3^ g^–1^ and the mean pore diameter was 1.3993 nm. According to the above-mentioned results, it can be deduced that the fabricated CA-PEG blend membrane is porous with micropores^40–43^.

The MP-plot is convenient for defining the presence or absence of micropores and their range of size^40–43^. From the Fig. 7c, it can be deduced that the fabricated CA-PEG blend membrane has micropores diameter (*d*_p_) of 0.7 to 0.9 nm with a distribution peak at 0.8 nm. Also, it has micropores diameter of 1.1 to 1.25, 1.25 to 1.4, 1.4 to 1.55 and 1.6 to 1.8 nm with distribution peaks at 1.2, 1.3, 1.5 and 1.7 nm, respectively. The total specific surface area (*a*_1_) was 0.5349) m^2^ g^–1^, the external specific surface area (*a*_2_) was 5.4092 m^2^ g^–1^, and the pore volume (*V*_p_) was 0.0009 cm^3^ g^–1^.

#### X-ray diffraction (XRD) analysis

**Figure 8:**
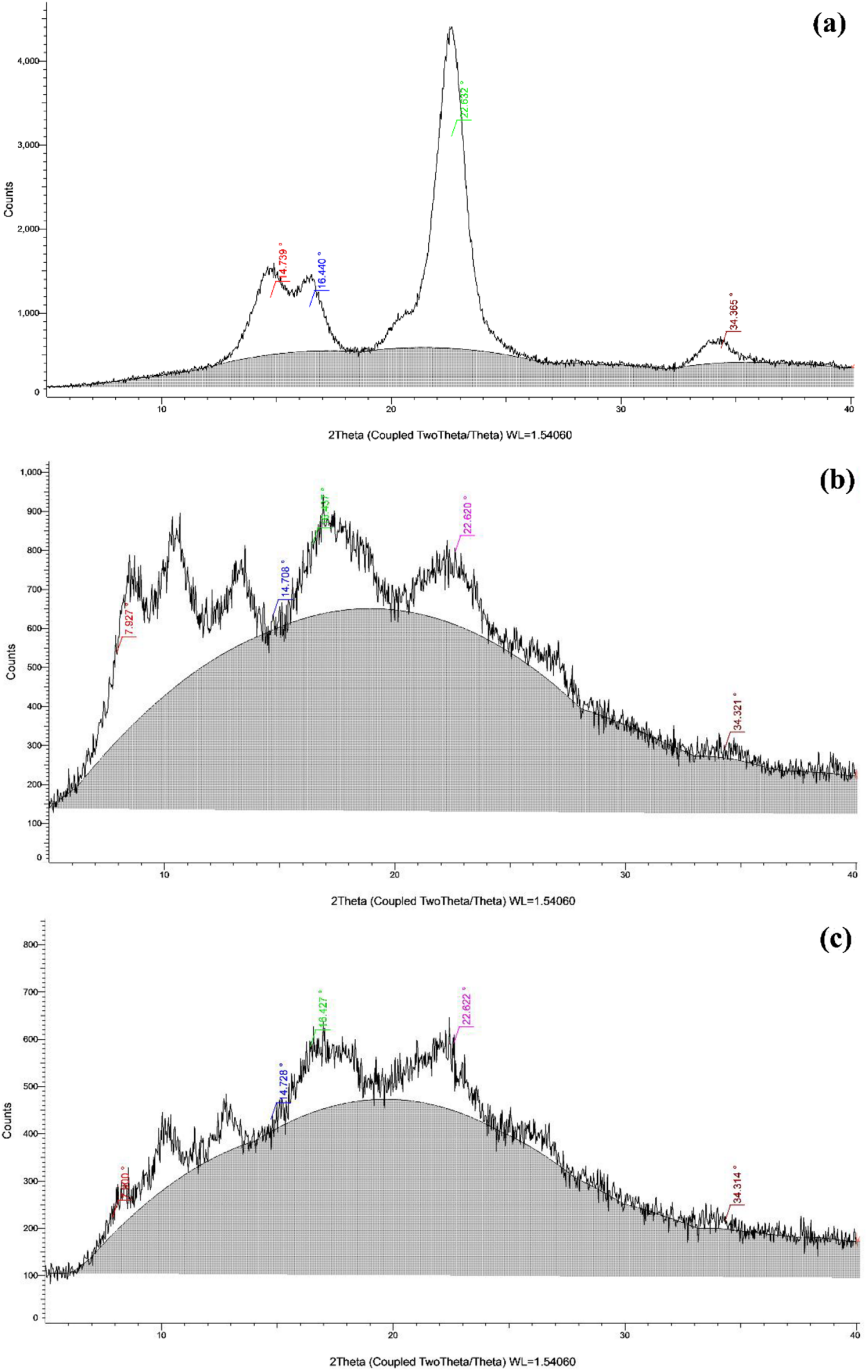
XRD analysis of (**a**) Cellulose extracted from cotton Giza 86, (**b**) Cellulose di-acetate, (c) CA-PEG blend membrane.

The XRD analysis was used to confirm the occurrence of a targeted successful acetylation process. It is known that the attendance of the acetyl groups in cellulose increased the hydrophobicity and increased the hydrophobicity of the prepared membrane. The patterns of cellulose extracted from cotton Giza 86, the cellulose acetate (Sample 5) and the fabricated CA-PEG blend membrane are compared in Fig. 8a,b,c. Figure 8a gave X–ray patterns with major peaks at 14.7°(I*α* (100) or I*β* (110)), 16.4° (I*α* (010) or I*β* (110)), 22.8° (I*α* (110) or I*β* (200)) and 34.4° ((I*α* and I*β* (400)), reflections for cellulose extracted from cotton Giza 86, which may be belongs to the I*α* or I*β* predominant. The *Z* function Eq. (6) was used to determine the conformation of the chiral carbon of cellulose.

6 $$Z=1693 d1-902 d2-549$$

The *d*1 was found to be 6.005 nm (*d*-spacing of peak at 14.7°), *d*2 was 16.504 nm (*d*-spacing of the peak at 16.4° (2*θ*), and *Z* value was –5268.474. From the negative *Z*-value of cellulose extracted from cotton Giza 86, it can be deduced that the extracted cellulose compound has I*β* dominant form^44^. Figure 8b,c gave X–ray patterns of the cellulose acetate (Sample 5) and the fabricated CA-PEG blend membrane with the same previous major peaks, but the relative height of intensity of diffraction peaks was decreased while the amorphous region increased. Also, there was a broadening in diffraction peaks occurred. The degree of crystallinity of the cellulose sample was considered to be 45.6%, the cellulose acetate (Sample 5) was 22.5% while in the fabricated CA-PEG blend membrane was 15.2%, which indicated a decrease in the crystallinity degree after esterification. This decrease in crystallinity in cellulose acetate (Sample 5) and the fabricated CA-PEG blend membrane compared with the cellulose sample could be due to the replacement of the hydroxyl groups by acetyl groups with a larger volume. During acetylation, the acetyl group was causing the breaking of inter- and intramolecular hydrogen bonds of cellulose, leading to the degradation in the crystal structure of cellulose. This result confirmed why the cellulose acetate (Sample 5) and the fabricated CA-PEG blend membrane are highly amorphous^45^. Also, Fig. 8b,c showed a remarkable peak at 7.9° (2*θ*) assigned to semicrystalline acetylated cellulose. This result was attributed to the presence of acetyl groups which caused an increase in the interfibrillar distance of the cellulose chains^46,47^.

#### Scanning electron microscope (SEM)

**Figure 9:**
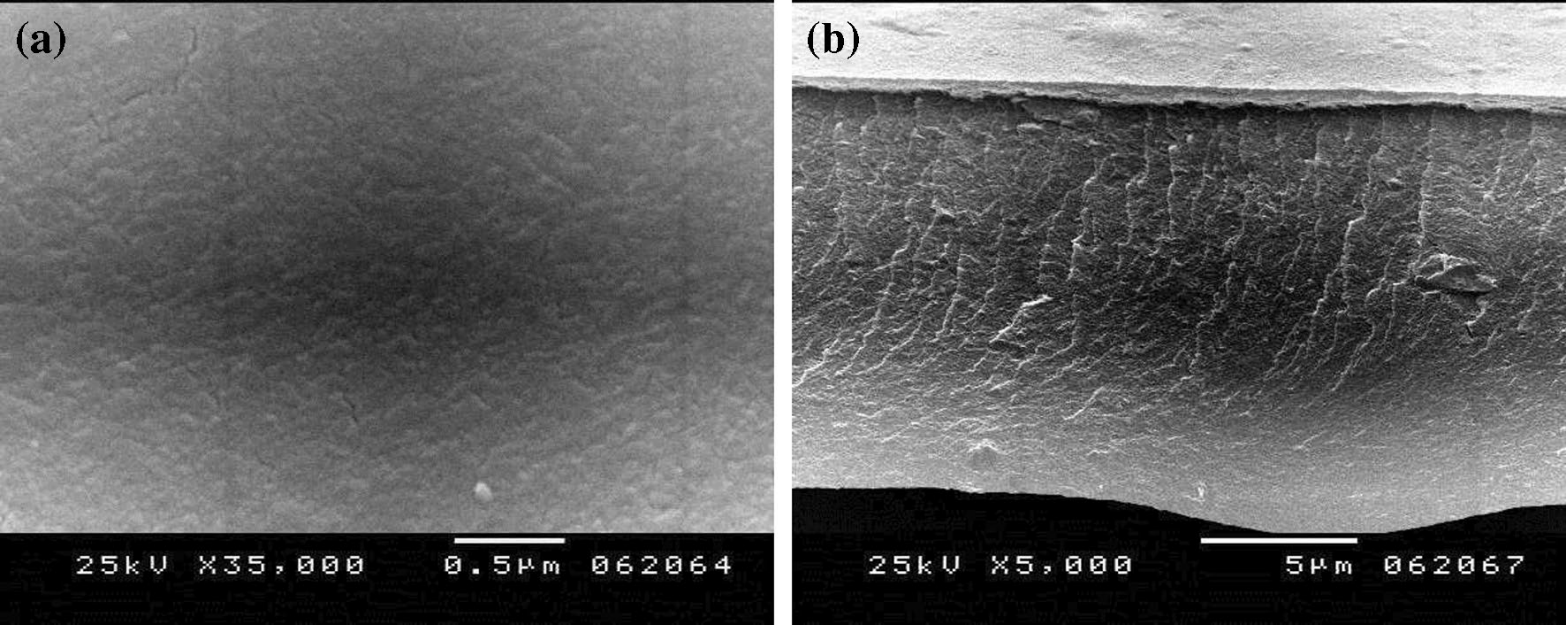
SEM analysis of CA-PEG blend membrane (**a**) Surface morphology, and (**b**) Cross-sectional morphology.

Figure 9 shows the SEM surface and cross-sectional morphology of the fabricated CA-PEG blend membrane. From Fig. 9, the creation of an asymmetric membrane with nano-sized pores can be clearly viewed. The blending of PEG with cellulose acetate during the casting process improved the phase inversion mechanism.

#### Water adsorption measurement

Water absorption measurement was used to define the hydrophobicity and hydrophilicity of the prepared membrane. According to Eq. (2), the percentage of water absorption of fabricated CA-PEG blend membrane was found to be 2.91%. The very lowering value of water absorption measurement indicated that the fabricated CA-PEG blend membrane is highly hydrophobic.

#### Contact angle measurement

The contact angle was also used to characterize the hydrophobicity and hydrophilicity of the membrane, it is known that a high contact angle value usually means high hydrophobicity. The fabricated CA-PEG blend membrane showed that, the value of the contact angle was 92.5 degree. The increment of contact angle value means, the fabricated CA-PEG blend membrane is highly hydrophobic^48,49^.

#### Porosity percentage measurement

The porosity of the fabricated CA-PEG blend membrane was calculated according to Eq. (3), and it was found to be 1.22%. The membrane porosity was enhanced when the PEG was blending with cellulose acetate. This value indicated that the fabricated membrane could be used for an ultrafiltration application (0.1–10%)^50^.

### The pure water permeation flux (PWP)

The flux of pure water is the most parameter used to measure the hydraulic permeability of the membrane in micro, dialysis and ultrafiltration membranes. The flux of Pure water of fabricated CA-PEG blend membrane was calculated by the dead-end filtration test cell according to Eq. (3) by determining the amount of the pure collecting water (0.078 L) through a certain time (3.5 h) under a certain pressure (1.0–2.0 bar)^51–54^. Distilled water is used as the feed in the flux performance experiment. In fabricated CA-PEG blend membrane, the pure water permeation flux (PWP) value at the operating pressure (1.0–2.0 bar) was 17.73 L/m^2^∙h. From this value, we can deduced that the water flux performance of the fabricated CA-PEG blend membrane from synthesized cellulose di-acetate (Sample 5) by using NiCl_2_ as a catalyst was convenient to use in the dialysis process^51–54^.

### BSA Rejection performance

Bovine serum albumin has an important role during the formation of calcium oxalate crystals which prevents the nucleation of calcium oxalate, so the urine stone will not be formed. From the view of the importance of bovine serum albumin, it is necessary not to lose the patient BSA during the dialysis process. The result in terms of BSA rejection indicates that the fabricated CA-PEG blend membrane from synthesized cellulose di-acetate (Sample 5) can retain all of the BSA successfully and with the highest rate of 100%^55,56^.

### Urea clearance efficiency

Urea clearance is important to assess kidney functions, so urea clearance should be measured. Urea clearance was the amount of blood or plasma cleared by the kidneys from urea within minutes. According to Eq. (5), urea clearance percentage was 67.2% after 2.5 h for the fabricated CA-PEG blend membrane from synthesized cellulose di-acetate (Sample 5). All the above-mentioned results proved the applicability of the fabricated CA-PEG blend membrane for dialysis^57–59^.

## Conclusion

Synthesis of the cellulose di-acetate using a solvent-free acetylation system from cellulose extracted from cotton Giza 86 by investigation of 200 and 300 mL Ac_2_O in the existence of NiCl_2_^.^6HO (1.0, 1.5 and 2.0 g) as a new catalyst was completed using the conventional reflux and the microwave irradiation methods. The prepared cellulose di-acetate was tested for the fabrication of PEG blend membrane. The fabricated CA-PEG blend membrane was also tested for dialysis application. The preparation of cellulose di-acetate using the microwave irradiation method showed excellent yields and short reaction time, which makes the proposed method important for commercial trial. The impact of the two methods used on the formation of cellulose di-acetate and its use in the dialysis membrane formulations was studied. The experimental degree of substitution of the prepared cellulose di-acetate values was in agreement with that calculated by FTIR and ^1^H-NMR analysis methods. The fabricated membrane was found to be applicable in the dialysis operation regarding urea clearance, pure water flux and rejection of Bovine Serum Albumin (BSA). Certainly, the present work is promising and applicable in dialysis membranes.

## Data Availability

The datasets used in this investigation are accessible for review upon request from the corresponding author of the paper.

## List of symbols

AC: Activated carbon
CA: Cellulose acetate
Ac_2_O: Acetic anhydride
*a*_sBET_: BET specific surface area
BET: Brunauer–Emmett–Teller
Bi(OTf)_2_: Bismuth(III) trifluoromethanesulfonate
BSA: Bovine Serum Albumin
*C*: Energy constant of the first layer
CA-PEG: Cellulose acetate-polyethylene glycol
CoCl_2_: Cobalt chloride
CRT: Creatinine
Cu(OTf)_2_: Copper(II) trifluoromethanesulfonate
DP: Degree of polymerization
*d*_p_: Micropores diameter
DS: Degree of substitution
DTTB: 4,4′-Di-*tert*-butylbiphenyl
Dw: Distilled water
EtOH: Ethanol
EVAL: Ethylene vinyl alcohol
FA: Formic acid
FTIR: Fourier-transform infrared spectroscopy
GO: Graphene oxide
GPC: Gel Permeation Chromatograph
H_2_O_2_: Hydrogen peroxide
HP: Hemoperfusion
LiAlH_4_: Lithium aluminum hydride
MMMs: Mixed-matrix membranes
Mn: Number average molecular weight
MP: Micropore
Mp: Molecular weight at peak maximum of the elugram
MSG: Monosodium glutamate
Mv: Viscosity average molecular weight
Mw: Molecular weight
Mz: Average molecular weight
NaBH_4_: Sodium borohydride
NaOCl: Sodium hypochlorite
NiCl_2_^.^6HO: Nickel chloride six hydrate
NMR: Nuclear magnetic resonance
PAN: Polyacrylonitrile
PC: P-cresol
PD: Polydispersity index
PEG: Polyethylene glycol
PEI: Polyetyleneimine
PMMA: Polymethyl methacrylate
PS: Polysulfone
PVA: Polyvinyl alcohol
PWP: Pure water permeation flux
Sc(OTf)_3_: Scandium(III) triflate
SEM: Scanning electron microscope
Sn(OTf)_2_: Tin(II) trifluoromethanesulfonate
TGA: Thermo gravimetrical analysis
*V*_m_: Monolayer volume
*V*_p_: Pore volume
*V*_T_: Total pore volume
WG: Weight gain
XRD: X-ray diffraction analysis
ZnCl_2_: Zink chloride
ZO: Zeolite

## References

[1] El Nemr A, Ragab S, El Sikaily A, Khaled A (2015). Synthesis of cellulose triacetate from cotton cellulose by using NIS as a catalyst under mild reaction conditions. Carbohydr. Polym..

[2] El Nemr A, Ragab S, El Sikaily A (2017). Rapid synthesis of cellulose triacetate from cotton cellulose and its effect on specific surface area and particle size distribution. Iran. Polym. J..

[3] Ragab S, Eleryan A, El Nemr A (2022). Ferric perchlorate hydrate as a new catalyst for highly efficient esterification of cellulose at room temperature. Sci. Rep..

[4] Ragab S, El Nemr A (2019). Zirconyl chloride as a novel and efficient green Lewis acid catalyst for direct acetylation of cotton cellulose in the presence and absence of solvent. J. Polym. Res..

[5] El Nemr A, Ragab S (2018). Acetylation of Cotton-Giza 86 cellulose using MnCl_2_ as a new catalyst and its application to machine oil removal. Environ..

[6] Baker RH, Bordwell FG (1955). Organic Syntheses.

[7] Iqbal J, Srivastava RR (1992). Cobalt (II) chloride catalyzed acylation of alcohols with acetic anhydride: Scope and mechanism. J. Org. Chem..

[8] Ishihara K, Kubota M, Kurihara H, Yamamoto H (1996). Scandium trifluoromethanesulfonate as an extremely active Lewis acid catalyst in acylation of alcohols with acid anhydrides and mixed anhydrides. J. Org. Chem..

[9] Orita A, Tanahashi C, Kakuda A, Otera J (2001). Highly powerful and practical acylation of alcohols with acid anhydride catalyzed by Bi(OTf)_3_. J. Org. Chem..

[10] Chandra KL, Saravanan P, Singh RK, Singh VK (2002). Lewis acid catalyzed acylation reactions: Scope and limitations. Tetrahedron.

[11] Meshram G, Patil VD (2009). Simple and efficient method for acetylation of alcohols, phenols, amines, and thiols using anhydrous NiCl_2_ under solvent-free conditions. Synth. Commun..

[12] Lee SE, Vyle JS, Williams DM, Grasby JA (2000). Novel syntheses of (*Z*)-alkene and alkane base-modified nucleosides. Tetrahedron Lett..

[13] Alonso F, Radivoy G, Yus M (2003). Active nickel-based reduction of organic compounds. Russ. Chem. Bull..

[14] Alonso F, Yus M (2004). The NiCl_2_–Li–arene (cat.) combination: A versatile reducing mixture. Chem. Soc. Rev..

[15] Alonso F, Riente P, Yus M (2011). Nickel nanoparticles in hydrogen transfer reactions. Acc. Chem. Res..

[16] Alonso F, Yus M (1996). Hydrogenation of olefins with hydrated nickel chloride, lithium and a catalytic amount of naphthalene. Tetrahedron Lett..

[17] Alonso F, Yus M (1997). Hydrogenation of alkynes with hydrated nickel chloride, lithium and a catalytic amount of naphthalene. Tetrahedron Lett..

[18] Ibragimov AG, Ramazanov IR, Khalilov LM, Sultanov RM, Dzhemilev UM (1996). Regio-and stereo-selective hydroalumination of disubstituted acetylenes with Et3Al catalysed by (η_5_-C_5_H_5_) 2TiCl_2_. Mendeleev Commun..

[19] Loupy A, Varma RS (2006). Microwave effects in organic synthesis. Chim. Oggi–Chem. Today..

[20] Motasemi F, Ani FN (2012). A review on microwave-assisted production of biodiesel. Renew. Sustain. Energy Rev..

[21] Buchori L, Istadi I, Purwanto P (2016). Advanced chemical reactor technologies for biodiesel production from vegetable oils—A review. Bull. Chem. React. Eng..

[22] Nguyen HC, Ong HC, Pham TTT, Dinh TKK, Su C-H (2020). Microwave mediated noncatalytic synthesis of ethyl levulinate: A green process for fuel additive production. Int. J. Energy. Res..

[23] Constantinou-Kokotou V, Peristeraki A (2004). Microwave-assisted NiCl_2_ promoted acylation of alcohols. Synth. Commun..

[24] El Nemr A, Ragab S, El Sikaily A (2016). Testing zinc chloride as a new catalyst for direct synthesis of cellulose di-and tri-acetate in a solvent free system under microwave irradiation. Carbohydr. Polym..

[25] Ragab S, El Nemr A (2017). Nanofiber cellulose di- and tri-acetate using ferric chloride as a catalyst promoting highly efficient synthesis under microwave irradiation. J. Macromol. Sci. Pure Appl. Chem..

[26] Deshmukh SP, Li K (1998). Effect of ethanol composition in water coagulation bath on morphology of PVDF hollow fiber membrane. J. Membr. Sci..

[27] Loeb S, Sourirajan S (1963). Sea water demineralization by means of an osmotic membrane. Adv. Chem. Ser..

[28] Chen Y, Xiong XP, Yang GA, Zhang LN, Lei SL, Liang H (2002). Characterization of regenerated cellulose acetate. Chin. J. Polym. Sci..

[29] Combe C, Molis E, Lucas P, Riley R, Clark MM (1999). The effect of CA membrane properties on adsorptive fouling by humic acid. J. Membr. Sci..

[30] Chaudry MA (2002). Water and ions transport mechanism in hyperfiltration with symmetric cellulose acetate membranes. J. Membr. Sci..

[31] Yin, L., Huanlin, C., & Bogeng, L. Influence of additives on phase separation process of PVDF solution and membrane morphology. *Acta Polym Sin*. **5**, 656–661 (2002). https://doi.org/WOS:000178936800021

[32] Azhar O, Jahan Z, Sher F, Niazi MBK, Javed S, Shahid KM (2021). Cellulose acetate-polyvinyl alcohol blend hemodialysis membranes integrated with dialysis performance and high biocompatibility. Mater. Sci. Eng..

[33] Waheed H, Minhas FT, Hussain A (2018). Cellulose acetate/sericin blend membranes for use in dialysis. Polym. Bull..

[34] Waheed H, Hussain A (2019). Fabrication of cellulose acetate/polyaziridine blended flat sheet membranes for dialysis application. Bio Nanosci..

[35] Idris A, Yee HK, Kee CM (2009). Preparation of cellulose acetate dialysis membrane using D-glucose monohydrate as additive. J. Teknol..

[36] Idris A, Lee KY (2006). The effect of different molecular weight PEG additives on cellulose acetate asymmetric dialysis membrane performance. J. Membr. Sci..

[37] Kee CM, Idris A (2010). Permeability performance of different molecular weight cellulose acetate hemodialysis membrane. Sep. Purif. Technol..

[38] Hsiao Y-S, Tran HN, Ke J-W, Fu C-C, Syu W-L, Liu S-H, Juang R-S (2022). Porous cellulose acetate mixed-matrix membrane adsorbents for efficient clearance of p-cresol and creatinine from synthetic serum. J. Taiwan. Inst. Chem. Eng..

[39] Gornall AG, Bardawill CJ, David MM (1949). Determination of serum proteins by means of the biuret reaction. J. Biol. Chem..

[40] Rouquerol F, Rouquerol J, Sing KSW (1999). Adsorption by powders and porous solids.

[41] Gregg SJ, Sing KSW (1982). Adsorption surface area and porosity.

[42] Brunauer S, Emmett PH, Teller E (1938). Adsorption of gases in multimolecular layers. J. Am. Chem. Soc..

[43] Barrett EP, Joyner LG, Halenda PP (1951). The determination of pore volume and area distributions in porous substances I. Computations from nitrogen isotherms. J. Am. Chem. Soc..

[44] Hult EL, Iversen T, Sugiyama J (2003). Characterization of the supermolecular structure of cellulose in wood pulp fibres. Cellulose.

[45] Nabili A, Fattoum A, Christine M, Salon B, Bras J (2017). Synthesis of cellulose triacetate-I from microfibrillated date seeds cellulose (*Phoenix dactylifera* L.), Elimame Elaloui1. Iran. Polym. J..

[46] Hu W, Chen S, Xu Q, Wang H (2011). Solvent-free acetylation of bacterial cellulose under moderate conditions. Carbohyd Polym..

[47] Zepic V, Poljanšek I, Oven P, Škapin AS, Hancic A (2015). Effect of drying pretreatment on the acetylation of nanofibrillated cellulose. BioResources.

[48] Nurkhamidah S, Rahmawati Y, Gunardi I, Alifiyanti P, Priambodo KD, Zaim RL, Muqni WE (2018). Enhancing properties and performance of cellulose acetate/polyethylene glycol (CA/PEG) membrane with the addition of titanium dioxide (TiO_2_) by using surface coating method. MATEC Web Conf..

[49] Jayalakshmi A, Kim I-C, Kwon Y-N (2015). Cellulose acetate graft-(glycidylmethacrylate-*g*-PEG) for modification of AMC ultrafiltration membranes to mitigate organic fouling. RSC Adv..

[50] Cuperus FP, Smolders CA (1991). Characterization of UF membranes membrane characteristics and characterization techniques. Adv. Colloid Interface Sci..

[51] Singh R (2014). Membrane technology and engineering for water purification: application, systems design and operation.

[52] Jagschies G, Lindskog E, Lacki K, Galliher PM (2018). Biopharmaceutical processing: development, design, and implementation of manufacturing processes.

[53] Luis, P. *Fundamental Modeling of Membrane Systems: Membrane and Process Performance*. ISBN: 978-0-12-813483-2. (Elsevier Ltd, 2018). 10.1016/C2016-0-02489-0

[54] Basile, A., & Nunes, S. P. (Eds.). *Advanced Membrane Science and Technology for Sustainable Energy and Environmental Applications* (2011).

[55] Aronson, J.K., DPhil, M.A., FRCP, M.B.Ch.B., HonFFPM, HonFBPhS, Bovine serum albumin. in *Meyler's Side Effects of Drugs*, 1045–1045. 16th edn (Elsevier B.V., 2016)

[56] Jahanban-Esfahlan A, Ostadrahimi A, Jahanban-Esfahlan R, Roufegarinejad L, Tabibiazar M, Amarowicz R (2019). Recent developments in the detection of bovine serum albumin. Int. J. Biol. Macromol..

[57] Farnebo S, Samuelsson A, Henriksson J, Karlander L-E, Sjöberg F (2010). Urea clearance: A new method to register local changes in blood flow in rat skeletal muscle based on microdialysis. Clin. Physiol. Funct. Imaging.

[58] Carson RC, Kiaii M, MacRae JM (2005). Urea clearance in dysfunctional catheters is improved by reversing the line position despite increased access recirculation. Am. J. Kidney. Dis..

[59] Chin AI, Sheth V, Kim J, Bang H (2019). Estimating residual native kidney urea clearance in hemodialysis patients with and without 24-h urine volume. Kidney Med..

